# Non‐canonical binding of the *Chaetomium thermophilum* PolD4 N‐terminal PIP motif to PCNA involves Q‐pocket and compact 2‐fork plug interactions but no 3_10_ helix

**DOI:** 10.1111/febs.16590

**Published:** 2022-08-22

**Authors:** Dongxiao Yang, Magnus S. Alphey, Stuart A. MacNeill

**Affiliations:** ^1^ Biomedical Sciences Research Complex, School of Biology University of St Andrews UK

**Keywords:** *Chaetomium thermophilum*, DNA polymerase δ, DNA replication, PCNA, PolD4

## Abstract

DNA polymerase δ (Pol δ) is a key enzyme for the maintenance of genome integrity in eukaryotic cells, acting in concert with the sliding clamp processivity factor PCNA (proliferating cell nuclear antigen). Three of the four subunits of human Pol δ interact directly with the PCNA homotrimer via a short, conserved protein sequence known as a PCNA interacting protein (PIP) motif. Here, we describe the identification of a PIP motif located towards the N terminus of the PolD4 subunit of Pol δ (equivalent to human p12) from the thermophilic filamentous fungus *Chaetomium thermophilum* and present the X‐ray crystal structure of the corresponding peptide bound to PCNA at 2.45 Å. Like human p12, the fungal PolD4 PIP motif displays non‐canonical binding to PCNA. However, the structures of the human p12 and fungal PolD4 PIP motif peptides are quite distinct, with the fungal PolD4 PIP motif lacking the 3_10_ helical segment that characterises most previously identified PIP motifs. Instead, the fungal PolD4 PIP motif binds PCNA via conserved glutamine that inserts into the Q‐pocket on the surface of PCNA and with conserved leucine and phenylalanine sidechains forming a compact 2‐fork plug that inserts into the hydrophobic pocket on PCNA. Despite the unusual binding mode of the fungal PolD4, isothermal calorimetry (ITC) measurements show that its affinity for PCNA is similar to that of its human orthologue. These observations add to a growing body of information on how diverse proteins interact with PCNA and highlight how binding modes can vary significantly between orthologous PCNA partner proteins.

## Graphical abstract

The sliding clamp PCNA interacts with numerous protein partners by binding to a conserved PIP (PCNA interacting protein) motif. In this study, we describe the interaction of the N‐terminal PIP motif of the DNA polymerase δ subunit PolD4 from the thermophilic fungus *Chaetomium thermophilum* with its cognate PCNA, revealing a novel, non‐canonical binding mode in which the compact nature of the PIP precludes the formation of the more usual 3_10_ helix.

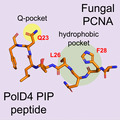

**Abbreviations**
BIR: break‐induced replication
HR: homologous recombination
IDCL: interdomain connector loop
ITC: isothermal titration calorimetry
MDPL: mandibular hypoplasia, deafness, progeroid features and lipodystrophy
PCNA: proliferating cell nuclear antigen
PIP: PCNA interacting protein
TDG: thymine DNA glycosylase

## Introduction

DNA polymerase δ (Pol δ) is a central enzyme for chromosomal DNA replication in eukaryotes, playing essential roles in both leading and lagging strand replication [1]. On the leading strand, Pol δ is responsible for initiating leading strand synthesis at replication origins, for recoupling polymerase and helicase activities during elongation and for the completion of leading strand synthesis during replication termination. On the lagging strand, Pol δ is responsible for the bulk of Okazaki fragment synthesis and also plays an important role in Okazaki fragment maturation [1]. Pol δ also plays important roles when replication forks stall or collapse during replication, playing key roles in break‐induced replication (BIR) and homologous recombination (HR)‐mediated replication restart, as well as in the repair of damaged DNA and recombination [2]. The importance of Pol δ for human health is illustrated by the fact that point mutations in Pol δ are a cause of MDPL (mandibular hypoplasia, deafness, progeroid features and lipodystrophy) syndrome [3, 4, 5] as well as a combined immunodeficiency syndrome [6, 7], and also predispose to a range of cancers [8, 9]. Copy number variations are frequently found in human cancers also [2].

Pol δ is a multi‐subunit complex, comprising a catalytic subunit with both polymerase and proofreading 3′ → 5′ exonuclease activities, and two or three accessory subunits. Human Pol δ is a tetrameric complex [10, 11, 12, 13] that consists of a catalytic subunit p125 (also termed PolD1) and accessory subunits p50 (PolD2), p66 (PolD3) and p12 (PolD4), whereas the budding yeast *Saccharomyces cerevisiae* Pol δ is a trimer of the p125, p50 and p66 orthologues Pol3, Pol31 and Pol32 respectively [14, 15, 16]. For maximal processivity, Pol δ acts in concert with the processivity factor PCNA (proliferating cell nuclear antigen) which is loaded onto DNA at the primer–template junction by replication factor C (RFC) and then acts as a sliding clamp, tethering the polymerase during DNA strand synthesis [17, 18]. PCNA is a homotrimer and on the surface of each protomer is found a hydrophobic pocket into which interaction partners dock through the use of a PCNA interacting protein (PIP) motif [19]. The canonical PIP motif has the sequence Qxxψxxθθ, where ψ and θ represent hydrophobic and aromatic residues, respectively, although an increasing number of non‐canonical PIP (or PIP‐like) motifs have been identified in recent years [20, 21].

**Fig. 1:**
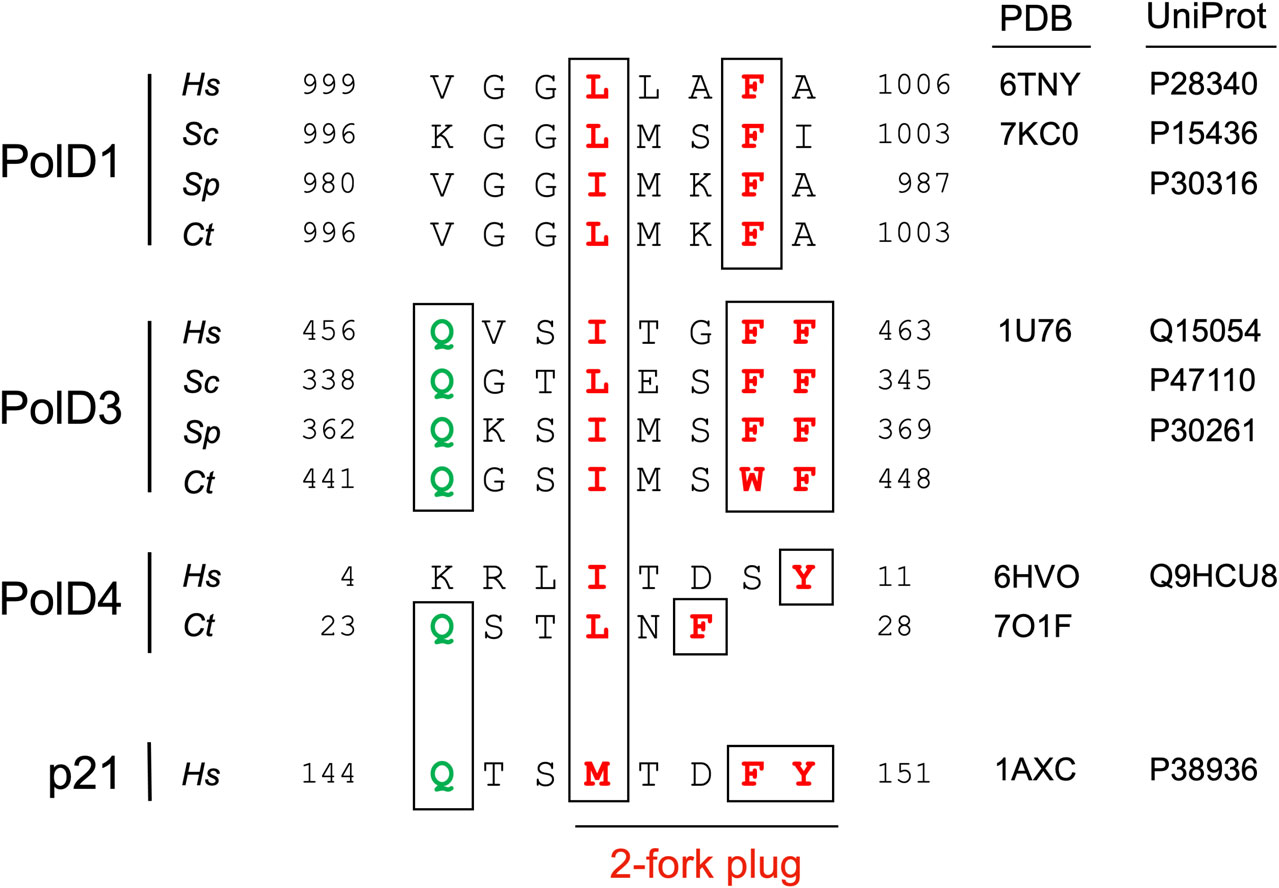
Known or predicted PIP motifs in DNA polymerase δ subunits from human (*Hs*), budding yeast *S. cerevisiae* (*Sc*), fission yeast *S. pombe* (*Sp*) and *Chaetomium thermophilum* (*Ct*). Conserved residues are shown in bold and either green (conserved glutamine that typically inserts in the PCNA Q‐pocket) or red (conserved hydrophobic and aromatic amino acids that insert into the PCNA hydrophobic pocket). The sequences were manually aligned. PDB and UniProt accession numbers are indicated on the right. The sequences of *Ct* PolD1 and *Ct* PolD3 (D. Yang and S. MacNeill, unpublished) are yet to be submitted to GenBank. The p21 PIP motif is shown below for comparison.

Three of the four subunits of human Pol δ contain PIP or PIP‐like motifs that have been shown to bind to PCNA (Fig. 1). The p66 (PolD3) PIP motif is located at the extreme C‐terminal end of the protein and has a canonical PIP motif sequence ^456^QVSITGFF^463^. In common with the archetypal PIP motif of the human p21^Cip1^ protein (^144^QTSMTDFY^151^), binding of the p66 PIP sees the insertion of the conserved glutamine 456 into the so‐called Q‐pocket on PCNA and the sidechains of the hydrophobic and aromatic residues isoleucine 459 and phenylamine 463, respectively, forming a 2‐fork plug that inserts into the hydrophobic pocket that lies under the interdomain connector loop (IDCL) on the surface of PCNA [22]. Similar to almost all structurally characterised PIP motifs, including p21^Cip1^, the central part of the PIP motif (^459^ITGF^462^) forms a 3_10_ helix. Canonical C‐terminal PIP motifs are a feature of the p66 (PolD3) orthologues Pol32 and Cdc27 from budding and fission yeasts [15, 23], respectively, suggesting a similar mode of binding to PCNA, although neither interaction has been characterised in atomic detail.

The human p12 (PolD4) PIP motif is located towards the N terminus of the protein and has the non‐canonical sequence ^4^KRLITDSY^11^ (Fig. 1) [24]. Structural analysis indicates that isoleucine 7 and tyrosine 11 are located in a 3_10_ helix and form a 2‐fork plug that inserts itself into the hydrophobic pocket on PCNA, while in the absence of the canonical conserved glutamine, the Q‐pocket is unoccupied [25].

The human p125 (PolD1) PIP motif is also non‐canonical and has the sequence ^1001^GLLAFA^1006^ (Fig. 1) [26]. In this case, leucine 1002 and phenylalanine 1005 form the 2‐fork plug and as with the binding of the human p12 PIP motif to PCNA, the Q‐pocket is unoccupied. Additional interactions between the thumb domain of p125 and the interdomain connector loop (IDCL) of PCNA contribute to the p125–PCNA interaction. This mode of PolD1–PCNA binding is also seen with the budding yeast Pol3 (PolD1) protein (PIP motif sequence ^998^GLMSFI^1003^) and PCNA [27].

Recent cryo‐EM studies of the human and yeast Pol δ–PCNA–DNA complexes have allowed Pol δ–PCNA interactions to be visualised for the first time in the context of the Pol δ holoenzyme (Pol δ–PCNA) [26, 27]. Strikingly, in both human and yeast Pol δ–PCNA–DNA structures, only the PolD1 (p125, Pol3) PIP motif is seen to interact with PCNA [26, 27], with the hydrophobic pockets on two of the three PCNA protomers being unoccupied. This observation suggests that the PolD3–PCNA and PolD4–PCNA interactions may not be a feature of the processive Pol δ holoenzyme, raising the question of what purpose these contacts serve.

The role of the PolD4 subunit in Pol δ function is also unclear [28, 29]. This protein is widely conserved in fungi, including the model fission yeast *Schizosaccharomyces pombe*, where the PolD4 orthologue, known as Cdm1, is non‐essential [30], but is absent from the budding yeast *S. cerevisiae* and related species. PolD4 orthologues are also found in animals [10] and plants, but the protein is not universally conserved across eukaryotic evolution (S. MacNeill, unpublished). The human PolD4 protein, p12, is non‐essential in cultured cells [31] and has further been shown to be degraded in response to DNA damage and upon entry into the S‐phase via the action of the cullin‐RING E3 ubiquitin ligase CRL4^Cdt2^ [32, 33] which targets key cell cycle proteins for proteolysis during S‐phase and after DNA damage [34]. The p12 PIP motif (^4^KRLITDSY^11^, described above) is classified as a PIP degron, a specialised form of the PIP motif that acts as a targeting signal for CRL4^Cdt2^ and subsequent degradation of the degron‐containing protein by the proteasome [35, 36]. PIP degrons are found in a range of proteins, many of which act in genome replication and repair, such as thymine DNA glycosylase (TDG), the histone methyltransferase Set8 and the DNA replication initiation factor Cdt1, as well as cell cycle regulators such as the *Drosophila* transcription factor E2f1 [34]. CRL4^Cdt2^‐mediated degradation of p12 leaves a three‐subunit Pol δ complex with enhanced proofreading ability [37] and altered properties in the processing of Okazaki fragments during lagging strand synthesis [38]. In the human Pol δ cryo‐EM structure, only the conserved C‐terminal domain of the p12 protein (residues 42–107) is visible, as a compact three‐helix bundle located at the interface of the p125 and p50 subunits [26]. Aside from the PIP motif (^4^KRLITDSY^11^), the N‐terminal region of the human p12 protein (residues 1–41) is predicted to be largely unstructured [25].

Understanding the diverse functions of Pol δ and how these functions are regulated in time and space presents a major challenge. In general, obtaining stable multi‐protein complexes with which to biochemically probe protein function can be difficult. In prokaryotic systems, one particularly productive approach has been to exploit the intrinsic enhanced stability of proteins native to thermophilic organisms [39]. Among eukaryotes, *Chaetomium thermophilum*, a thermophilic ascomycete fungus (phylum Ascomycota, subphylum Pezizomycotina) with an optimal growth temperature of 50–55 °C (maximum 60 °C), has been shown to have great potential in this regard [40]. Several recent studies have reported biochemical and structural analysis of multi‐protein complexes from *C. thermophilum*: examples include the inner ring of the nuclear pore complex (NPC) [41], the 80S ribosome [42] and head‐middle and tail modules of the mediator complex [43].

In order to gain insights into the structure, function and regulation of Pol δ, we have initiated a study of *C. thermophilum* Pol δ. Like human Pol δ, *C. thermophilum* Pol δ is a tetrameric complex, comprising orthologues of p125, p50, p66 and p12, designated PolD1, PolD2, PolD3 and PolD4 respectively (D. Yang and S. MacNeill, unpublished). Here, we describe the identification of *C. thermophilum* PolD4 and PCNA proteins, identify a candidate PIP motif in the N‐terminal region of PolD4, show that this is indeed capable of binding to *C. thermophilum* PCNA and determine the crystal structure of the PCNA–PIP peptide complex. The structure reveals a binding mode involving Q‐pocket and 2‐fork plug interactions, but with the compact nature of the PIP motif precluding the formation of a 3_10_ helix.

## Results

### Identification of *C. thermophilum* PolD4

To allow the development of a system for high‐level expression and purification of *C. thermophilum* Pol δ enzyme for structural studies, it was necessary to identify and characterise the genes encoding the Pol δ subunits in this organism and in particular, to experimentally confirm mRNA splicing patterns. BLAST (blastp) searching the NCBI non‐redundant protein collection (nr) protein sequences database using the 160 amino acid *S. pombe* Cdm1 protein as the query identified PolD4 homologues in two *Chaetomium* species (*Chaetomium* sp. MPI‐CAGE‐AT‐0009 and *C. globosum* CBS148.51) with 35–40% sequence identity over the ~ 70 amino acid conserved C‐terminal region of *S. pombe* Cdm1 (blastp *E*‐values of 6e‐15 and 3e‐14, respectively). Using the putative *C. globosum* PolD4 sequence for BLAST searching (tblastn) of the NCBI nucleotide collection (nr/nt) then led to the identification of a putative *C. thermophilum var. thermophilum* DSM1495 PolD4 protein with 46% identity to the *C. globosum* query (tblastn *E*‐value of 4e‐28). Subsequent cloning and sequencing of *C. thermophilum* cDNA led to confirmation that *C. thermophilum (Ct)* PolD4 is encoded by a 624 bp ORF that is interrupted by a single 102 bp intron sequence (see Table S1). *Ct* PolD4 is 208 amino acids in length (predicted M_r_ 23.3 kDa) and comprises an extended ~ 140 amino acid N‐terminal domain of limited sequence similarity to PolD4 proteins in other species (and predicted to be intrinsically disordered) and a conserved ~ 70 amino acid C‐terminal domain (data not shown).

### Identification and structure of *C. thermophilum* PCNA

**Fig. 2:**
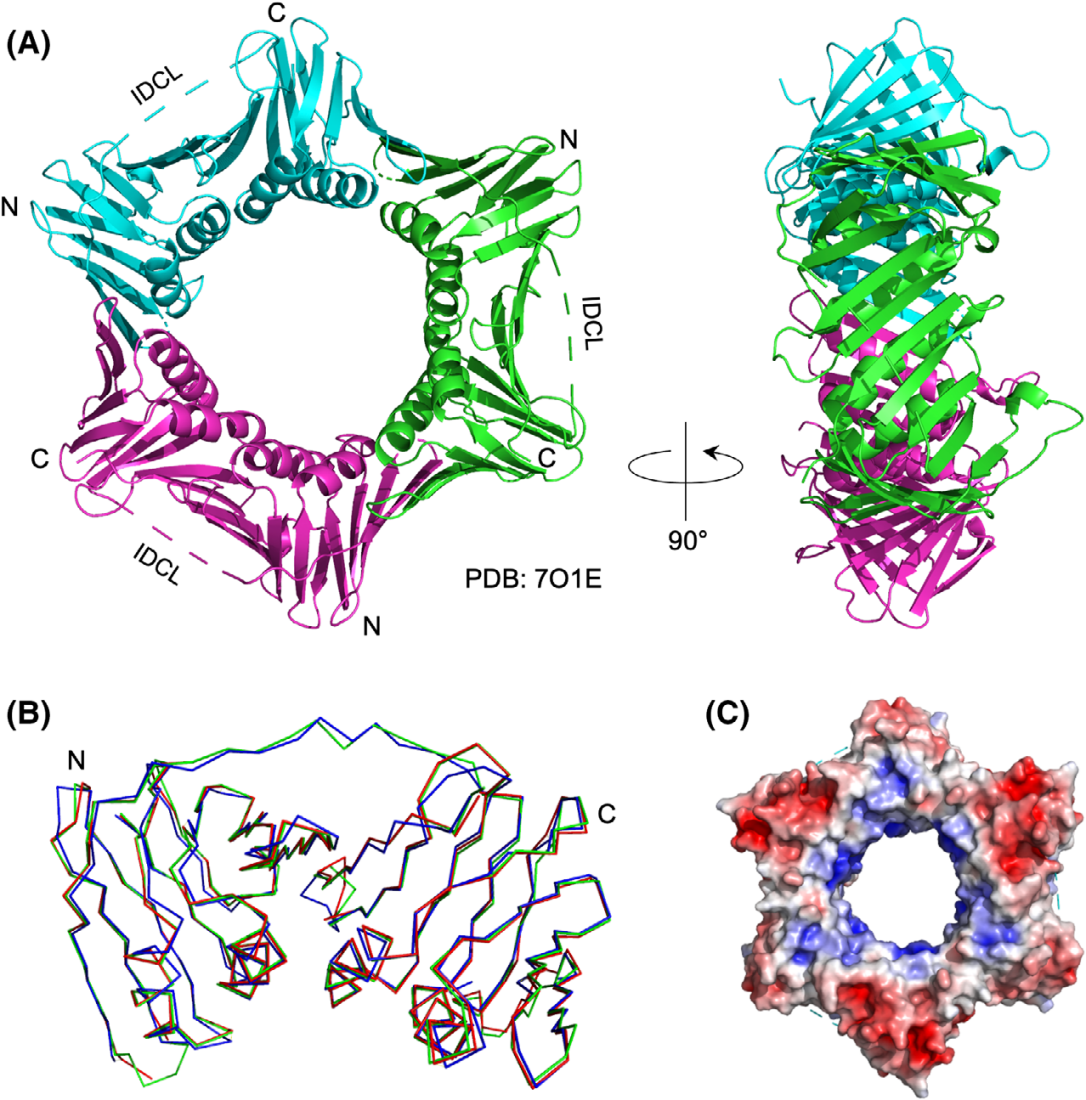
(A) Overall structure of *Ct* PCNA trimer at 2.34 Å resolution in front and side views, with individual protomers shown in green, cyan and magenta (PDB: 7O1E). (B) Superimposition of backbone traces from *C. thermophilum* (PDB: 7O1E, red), *N. crassa* (PDB: 7EP8, green) and human (PDB: 1AXC, blue) PCNA protomers, calculated using the align function in pymol. (C) Surface electrostatic potential of *Ct* PCNA contoured within a range −5 to +5 kT/e, calculated using the APBS electrostatics plugin in pymol. The red and blue colours denote negatively and positively charged surfaces respectively. Figure prepared using the pymol Molecular Graphic System, version 2.0.6 (Schrödinger, New York, NY, USA).

*Chaetomium thermophilum* PCNA was also identified by BLAST (tblastn) searching of the NCBI nucleotide collection (nr/nt) using *S. pombe* PCNA as the query (68% identity, *E*‐value 2e‐126). cDNA sequencing confirmed that *Ct* PCNA is encoded by a 777 bp ORF that is interrupted in the genome by two introns of 68 and 61 bp (see Table S2). *Ct* PCNA is a 259 amino acid protein (predicted M_r_ 28.6 kDa). The protein was expressed in recombinant form in *Escherichia coli*, purified to apparent homogeneity, concentrated to 14.4 mg·mL^−1^ and crystallised (see Materials and methods for details). The crystals diffracted to 2.34 Å with a single chain in the asymmetric unit. The structure was solved (Fig. 2A) by molecular replacement using a single chain from *Aspergillus fumigatus* PCNA (PDB: 5TUP) as a starting model [44] and shows high similarity to previously characterised PCNA structures, with RMSD values of 0.32 and 0.57 Å (calculated over 216 and 213 of 243 Cα atoms, respectively) to the fungus *Neurospora crassa* and human PCNAs (Fig. 2B). The trimeric assembly was generated by the crystallographic symmetry and displays characteristic features of PCNAs from other species, such as the positively charged central cavity through which DNA passes (Fig. 2C).

### Identification of a candidate PCNA interacting motif in *Ct* PolD4

**Fig. 3:**
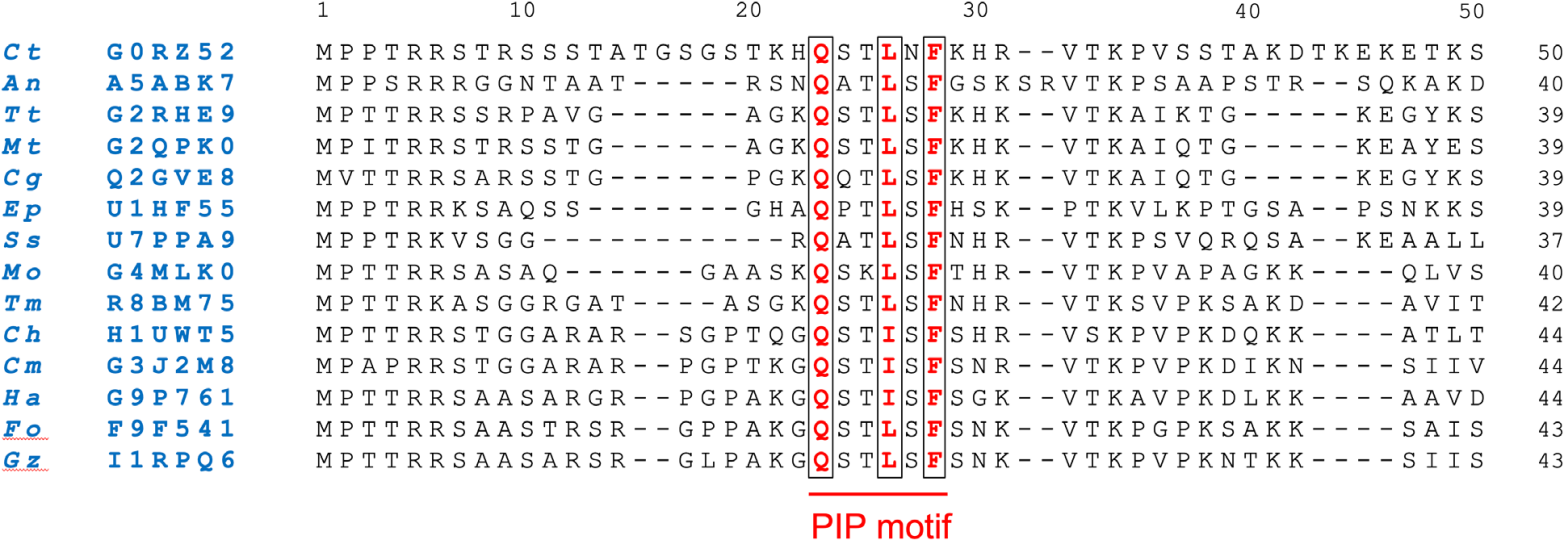
Multiple protein sequence alignment of the N‐terminal 50 amino acids of *Ct* PolD4 with the corresponding region from PolD4 orthologues (with 40–60% pairwise identity over the full length of the protein) from 13 related filamentous fungi (phylum Ascomycota, subphylum Pezizomycotina). The alignment was generated with Clustal Omega (EMBL‐EBI) using full‐length proteins and default alignment parameters. Conserved residues forming the putative PIP motif are shown in bold red and boxed. UniProt accession numbers are shown in blue to the left. Species abbreviations: *Ct* (*Chaetomium thermophilum*), *An* (*Aspergillus niger*), *Tt* (*Thermothielavioides terrestris*), *Mt* (*Myceliophthora thermophila*), *Cg* (*Chaetomium globosum*), *Ep* (*Endocarpon pusillum*), *Ss* (*Sporothrix schenckii*), *Mo* (*Magnaporthe oryzae*), *Tm* (*Togninia minima*), *Ch* (*Colletotrichum higginsianum*), *Cm* (*Cordyceps militaris*), *Ha* (*Hypocrea atroviridis*), *Fo* (*Fusarium oxysporum*) and *Gz* (*Gibberella zeae*).

In order to identify candidate PCNA interacting protein (PIP) motifs in the N‐terminal region of *Ct* PolD4, the protein sequence was aligned with its orthologues from diverse filamentous fungal species (Fig. 3). This identified a conserved sequence motif (QxxLxF) spanning amino acids 23–28 that resembles the archetypal PIP motif (Qxxψxxθθ, where ψ and θ represent amino acids with hydrophobic and aromatic side chains, respectively) but which differs from the archetypal PIP motif in two respects: only one aromatic residue (θ) is present (Phe28 in *Ct* PolD4) and the spacing between the central hydrophobic amino acid (Leu26 in *Ct* PolD4) and the aromatic residue (Phe28) is reduced to a single amino acid only, likely precluding the formation of a 3_10_ helix (see below).

**Fig. 4:**
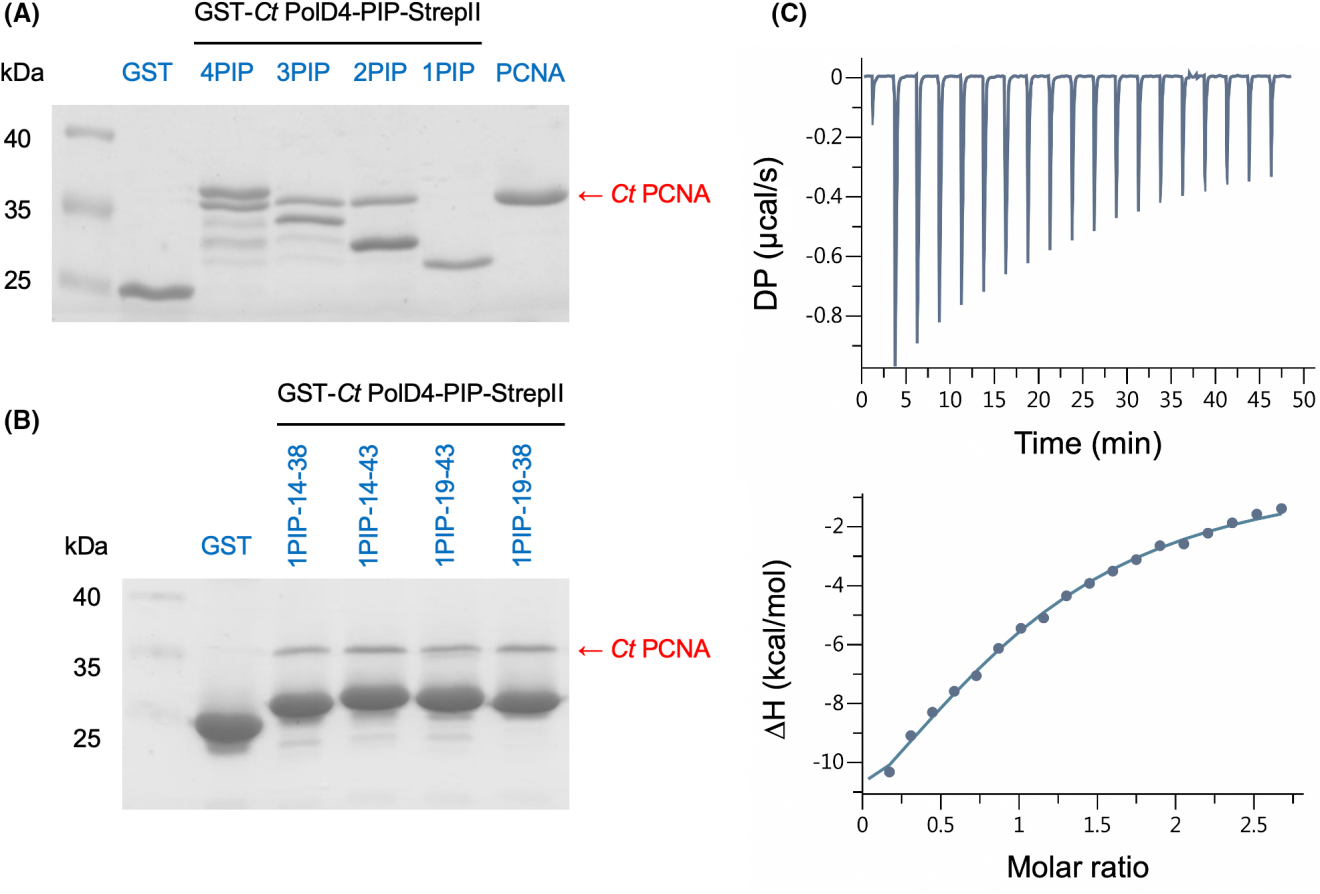
(A) PCNA pull‐down assays performed with GST and GST‐*Ct* PolD4‐PIP‐StrepII proteins carrying 1–4 copies of the *Ct* PolD4 PIP motif (amino acids 19–38). Bound proteins were visualised by PAGE Blue G90 staining following SDS/PAGE. PCNA binding is seen with 4PIP, 3PIP and 2PIP constructs only. (B) PCNA pull‐down assays were performed with GST and GST‐*Ct* PolD4‐PIP‐StrepII proteins carrying a single copy of the PIP motif flanked by additional amino acids (amino acids 14–38, 19–43 and 14–43). Sub‐stoichiometric but specific PCNA binding is seen with all four GST‐*Ct* PolD4‐PIP‐StrepII proteins, but no enhancement of binding is seen when the additional flanking amino acids are present. The pull‐down assays in Fig. 4A,B were each performed three times, with indistinguishable results; single representative gels are shown. (C) Affinity of a *Ct* PolD4 PIP peptide (^1^KHQSTLNFKHRVTKP^35^) for *Ct* PCNA measured by isothermal titration calorimetry. The upper panel shows baseline‐corrected experimental data from titration of the *Ct* PolD4 peptide (400 μm) with PCNA (30 μm). The lower panel shows the ligand concentration dependence of heat released upon binding. Molar ratio corresponds to peptide:PCNA protomer. The ITC analysis was performed twice with indistinguishable results; a single dataset is shown. See Materials and methods for details.

To test whether the candidate *Ct* PolD4 motif could bind to PCNA, various GST‐*Ct* PolD4‐PIP‐StrepII fusion proteins comprising single or multiple repeats of the putative PIP motif (amino acids 19–38 of *Ct* PolD4) were expressed in *E. coli* and purified (see Materials and methods). The purified proteins were then tested for their ability to pull down purified recombinant *Ct* PCNA (Fig. 4A). In initial experiments, efficient pull down of PCNA was seen with proteins carrying two, three or four tandem repeats of the putative PIP motif (Fig. 4A, lanes labelled 4PIP, 3PIP and 2PIP) but not with GST alone or with a GST fusion protein carrying a single PIP motif (1PIP). Increasing the amount of the 1PIP protein used for the pull downs resulted in weak yet specific PCNA binding (Fig. 4B, compare lanes labelled GST and 1PIP‐19‐38). The reason for the relatively weak binding of the single PIP motif GST fusion is not clear but could be due to steric hindrance by the GST moiety, although extending the single PIP motif by five residues at the N‐terminal end (amino acids 14–38), the C‐terminal end (amino acids 19–43) or at both ends (amino acids 14–43), in an effort to alleviate steric hindrance, did not significantly enhance *Ct* PCNA binding (Fig. 4B, lanes labelled 1PIP‐14‐38, 1PIP‐19‐43 and 1PIP‐14‐43).

### Binding affinity and stoichiometry

To examine the interaction between the *Ct* PolD4 PIP motif and *Ct* PCNA in greater detail and in the absence of GST, isothermal titration calorimetry (ITC) was used to determine the binding affinity of PCNA for a 15 amino acid synthetic PolD4 PIP peptide (sequence: ^21^KHQSTLNFKHRVTKP^35^) and the stoichiometry of the interaction (Fig. 4C). The dissociation constant (*K*_D_) was determined to be 22 ± 0.6 μm at 25 °C, broadly similar to that reported for the human PCNA–p12 PIP peptide interaction (38 ± 4 μm) [25]. The stoichiometry was determined to be 1 : 1 (i.e. 1 peptide : 1 PCNA protomer) indicating that the PCNA trimer is capable of binding three PIP peptides, as expected.

### Crystal structure of the PolD4 PIP peptide–PCNA complex

**Fig. 5:**
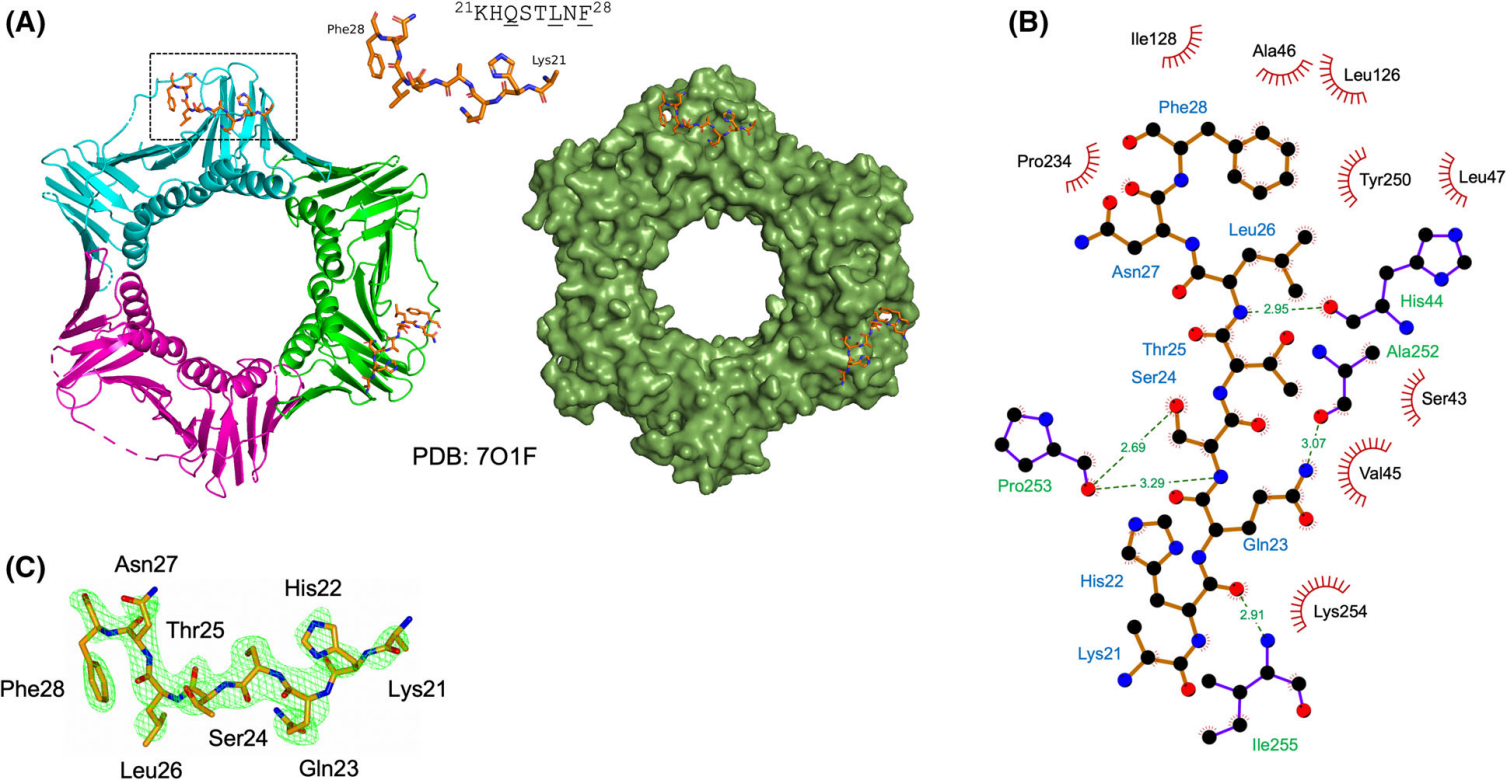
(A) Crystal structure of *Ct* PCNA with *Ct* PolD4 PIP peptides binding to two PCNA protomers at 2.45 Å (PDB: 7O1F). Only 8 of the 20 amino acids of the peptide are visible in the structure (^21^KHQSTLNF^28^, sequence shown with conserved PIP motif residues underlined). Figure prepared using the pymol Molecular Graphic System, version 2.0.6 (Schrödinger). (B) Detailed view of *Ct* PCNA–*Ct* PolD4 PIP peptide interaction visualised using LigPlot+ [57]. The *Ct* PolD4 peptide backbone is shown in brown, with individual peptide amino acids being labelled in blue text. Hydrogen bonds are shown as green broken lines, with distances marked. Amino acids in *Ct* PCNA involved in hydrogen bonding are labelled in green text. The red spoked arcs represent residues in *Ct* PCNA making hydrophobic contacts with the peptide – the corresponding atoms in the *Ct* PolD4 peptide are decorated with smaller red spokes. (C) Fo−Fc difference density map calculated (ligand removed, model re‐refined) for one of the *Ct* PolD4 PIP motif peptides bound to *Ct* PCNA. The difference in electron density, depicted as green mesh, is contoured at 2.5 σ. The CtPolD4 PIP peptide is shown as a stick model with atoms coloured according to type: carbon, orange; oxygen, red; and nitrogen, blue.

**Fig. 6:**
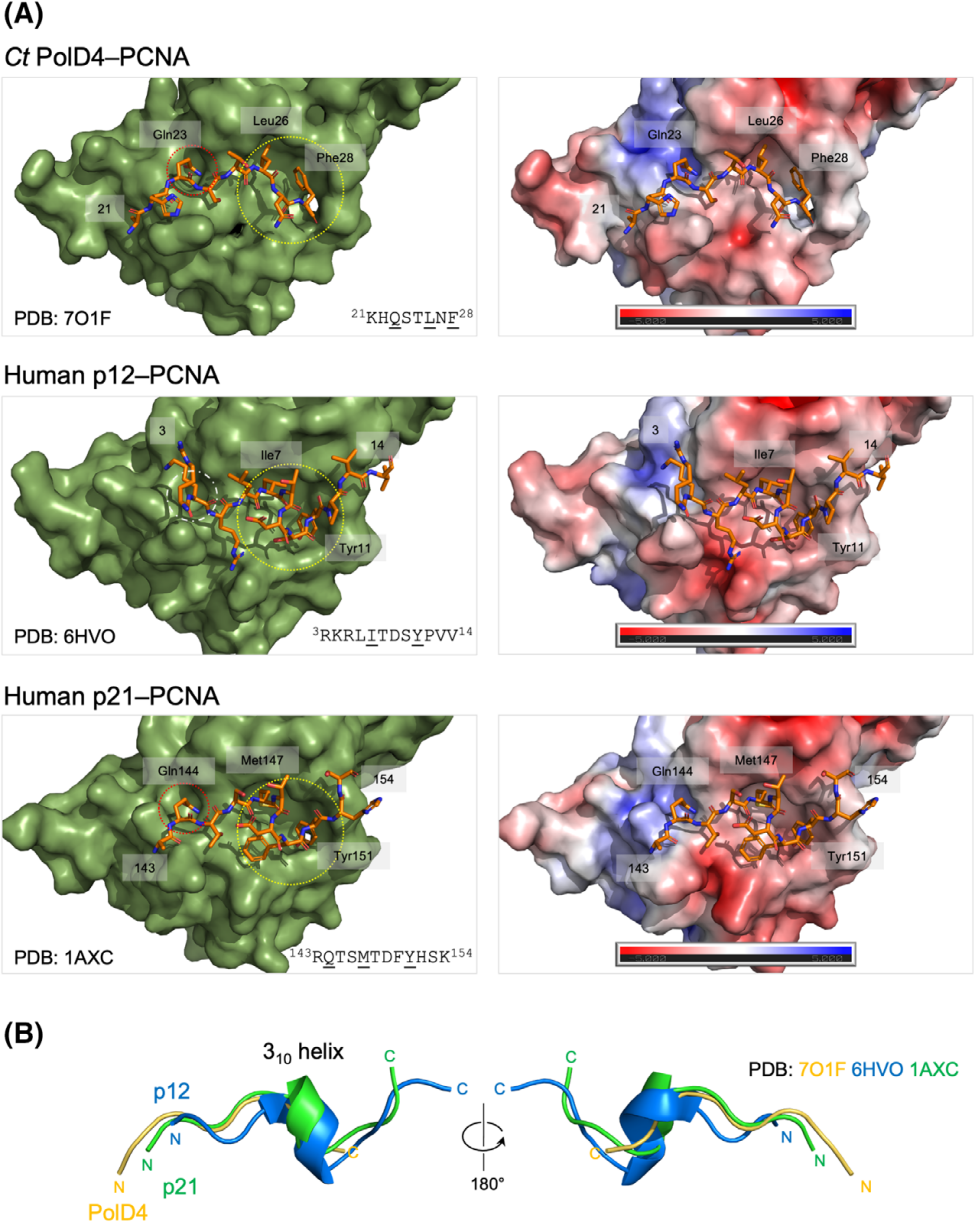
Comparison of *Ct* PolD4, human p12 and human p21^Cip1^ PIP peptide‐binding modes. (A) Upper left panel: Binding of *Ct* PolD4 peptide (^21^KHQSTLNF^28^, sequence shown below with conserved PIP motif residues underlined) to *Ct* PCNA protomer (PDB: 7O1F). The Q‐pocket (occupied by the sidechain of Gln23) is circled in red, and the hydrophobic pocket (occupied by the sidechains of Leu26 and Phe28) in yellow. Centre left panel: Binding of human p12 peptide (^3^RKRLITDSYPVV^14^, sequence shown below with conserved PIP motif residues underlined) to human PCNA protomer (PDB: 6HVO). The Q‐pocket (unoccupied) is circled in orange, and the hydrophobic pocket (occupied by the sidechains of Ile7 and Tyr11) in yellow. Lower left panel: Binding of human p21^Cip1^ peptide (^143^RQTSMTDFYHSK^154^, sequence shown below with conserved PIP motif residues underlined) to human PCNA protomer (PDB: 1AXC). The Q‐pocket (occupied by the sidechain of Gln144) is circled in red, and the hydrophobic pocket (occupied by the sidechains of Met147 and Tyr151) in yellow. Surface charge representations of all three binding modes calculated using the APBS Electrostatics plugin in pymol are shown to the right. The red and blue colours denote negatively and positively charged surfaces respectively. (B) Overlain ribbon diagrams of the *Ct* PolD4, human p12 and human p21^Cip1^ PIP peptides illustrating the presence/absence of the 3_10_ helix. Figure prepared using the pymol Molecular Graphic System, version 2.0.6 (Schrödinger).

To further characterise the binding of *Ct* PolD4 with *Ct* PCNA, and to allow detailed comparison with the human p12‐PCNA interaction, the structure of the *Ct* PolD4 PIP peptide–*Ct* PCNA complex was solved to a resolution of 2.45 Å (Table 1) with peptides seen to occupy two of three possible PIP motif binding sites on the PCNA trimer only (Fig. 5A), possibly due to occlusion of the third binding site in the packed crystals. Only the first eight of the 15 residues of the *Ct* PolD4 peptide (Lys21 to Phe28 in *Ct* PolD4) are visible in the electron density map (Fig. 5A). The conserved glutamine Gln23 inserts into the Q‐pocket on PCNA, with a hydrogen bond forming between Gln23 (NE2) in *Ct* PolD4 and Ala252 (O) in *Ct* PCNA (3.07 Å distance) and hydrophobic interactions between Gln23 and the sidechains of Val45 and Lys254 (see Figs 5B and 6A, upper left panel). Human p12 lacks the conserved glutamine, leaving the Q‐pocket unoccupied (Fig. 6A, compare upper and middle left panels) [25]. The sidechains of the central hydrophobic residue Leu26 and the single aromatic residue Phe28 in *Ct* PolD4 form a 2‐fork plug that inserts into the hydrophobic pocket located under the interdomain connector loop (IDCL), with interactions between Leu26 in *Ct* PolD4 and the sidechains of Leu47, Pro234 and Ala252 and the backbone groups of Val45 and Ala46 in PCNA, and between Phe28 in *Ct* PolD4 and the sidechains of Leu47, Leu126, Ile128 and Tyr250 in *Ct* PCNA (Fig. 5B). These interactions are similar to those seen with the human p12 PIP peptide, where Ile7 and Tyr11 insert into the hydrophobic pocket on PCNA (Fig. 6A, middle left panel), however, the short distance between the Leu26 and Phe28 in *Ct* PolD4 (one amino acid, rather than the three seen with human p12) does not allow formation of the 3_10_ helix seen in p12 [25] and other canonical PIP motifs including the human p21 archetype [45] (Fig. 6B). Additional hydrogen bonding interactions are seen between Ser24 (OG) in *Ct* PolD4 and Pro253 (O) in *Ct* PCNA (2.69 Å), Ser24 (N) in *Ct* PolD4 and Pro253 (O) in *Ct* PCNA (3.29 Å), His22 (O) in *Ct* PolD4 and Ile255 (N) in *Ct* PCNA (2.91 Å), and Leu26 (N) in *Ct* PolD4 and His44 (O) in *Ct* PCNA (2.95 Å). The latter H‐bond is conserved in the human p12 PIP motif–PCNA structure, linking Leu6 (O) in p12 with His44 (N) in PCNA, but the others are not.

**Table 1:** Data collection and refinement statistics.

	PCNA	PCNA–peptide complex
Data Collection		
PDB ID	7O1E	7O1F
Space group	H3	P1
Cell dimensions		
*a*,*b*,*c* (Å)	86.27, 86.27, 90.84	71.89, 84.71, 84.89
α,β,γ (°)	90.00, 90.00, 120.00	60.94, 89.60, 81.22
Resolution (Å)	30.28–2.34 (2.42–2.34)	30.39–2.45 (2.52–2.45)
*R*_merge_	0.097 (0.547)	0.130 (1.368)
*I*/σ*I*	9.6 (2.0)	7.2 (0.8)
Completeness (%)	98.9 (90.7)	94.2 (92.1)
Redundancy	5.2 (3.7)	3.9 (3.6)
CC ½	0.997 (0.786)	0.994 (0.406)
Refinement		
Resolution (Å)	30.28–2.34	30.39–2.45
No. reflections	9986	56 897
*R*_work_/*R*_free_ (%)	21.27/27.98	22.02/26.19
No. atoms		
Protein	1857	11 192
Ligand/ion	0/0	243/18
Water	42	160
B‐factors		
Protein	49.93	48.97
Ligand/ion	0/0	56.43/46.95
Water	45.12	43.14
RMSD		
Bond lengths (Å)	0.006	0.007
Bond angles (°)	1.444	1.461

## Discussion

DNA polymerase δ is a key player in the maintenance of genome integrity in eukaryotic cells, playing key roles in DNA replication, repair and recombination [1, 2]. Pol δ activity is essential for genome duplication, and mutations (or copy number variations) in Pol δ have been shown to underlie MDPL (mandibular hypoplasia, deafness, progeroid features and lipodystrophy) and combined immunodeficiency syndromes, as to predispose to a range of human cancers [3, 4, 5, 6, 7, 8, 9]. In mouse models, single amino acid changes in the catalytic subunit affecting Pol δ proofreading ability lead to the formation of a variety of tumour types [46, 47, 48].

Human Pol δ comprises four subunits: p125, p50, p66 and p12 (alternatively known as PolD1, PolD2, PolD3 and PolD4) [10, 11, 12, 13]. Two of the four subunits (p50 and p66) are also components of the translesion synthesis (TLS) polymerase Pol ζ [49]. Crucial for Pol δ activity is its ability to interact with the processivity factor PCNA. PCNA is a toroidal trimer that encircles double‐stranded DNA and acts to tether Pol δ to its substrate, thus conferring high processivity on the polymerase [20, 21]. PCNA also acts as a landing pad for numerous other factors involved in replication and repair.

There are at least three PCNA interaction sites in human Pol δ, in the p125, p66 and p12 proteins and possibly more. Each interaction site comprises a short, conserved sequence called a PIP or PIP‐like motif (for PCNA interacting protein) with consensus sequence Qxxψxxθθ, where ψ and θ represent amino acids with hydrophobic and aromatic sidechains respectively. Previous studies have characterised a range of PCNA–PIP motif interactions in atomic detail, including interactions involving the p66 and p12 subunits [22, 25]. The p12 PIP motif has a specialised function as a PIP degron, a targeting signal for the CRL4^Cdt2^ E3 ubiquitin ligase [35, 36]. CRL4^Cdt2^ ubiquitylates p12, leading to its proteasomal degradation, in response to DNA damage and upon entry into S‐phase [32, 33]. The three‐subunit Pol δ complex that remains (p125‐p50‐p66) has enhanced proofreading ability and altered properties in Okazaki fragment processing [37, 38].

As part of a study aimed at dissecting the structure, function and regulation of Pol δ, we have developed methods for high‐level expression and rapid purification of three‐ and four‐subunit Pol δ complexes and PCNA from the thermophilic fungal species *C. thermophilum* (D. Yang and S. MacNeill, unpublished). In this report, we describe the crystal structure of the *C. thermophilum* PCNA (*Ct* PCNA) and characterise its interaction with an N‐terminal PIP motif from the PolD4 subunit of *C. thermophilum* Pol δ using biochemical and structural methods. While the structure of the PolD4 PIP peptide–PCNA complex is broadly comparable to that of previously characterised PIP–PCNA pairings, several features are worthy of note.

Three amino acids are key to interactions between *Ct* PolD4 and *Ct* PCNA: Gln23, Leu26 and Phe28. All three are strictly conserved in PolD4 proteins from fungal species closely related to *C. thermophilum* (Fig. 3). The sidechain of Gln23 inserts into the Q‐pocket on PCNA while the sidechains of Leu26 and Phe28 insert into the hydrophobic pocket located under the interdomain connector loop (IDCL) on the surface of PCNA (Figs 5 and 6A, upper left panel). The human p12 protein lacks the glutamine, equivalent to Gln23, that marks the start of the canonical PIP motif (Qxxψxxθθ), instead having a lysine at the corresponding position (Lys4) that is unable to insert into the Q‐pocket (Fig. 6A, middle left panel) [25]. The absence of the glutamine is not uncommon among diverse PIP and PIP‐like motifs [21], indicating that insertion of glutamine into the Q‐pocket is not an absolute requirement for PIP motif binding, but its presence can contribute markedly to the affinity of binding. A recent analysis of the human p21^Cip1^ PIP motif (^144^QTSMTDFY^151^) in which the glutamine 144 was substituted with serine, methionine, lysine, aspartate or asparagine saw PCNA binding affinity (*K*_D_) reduced by 58‐ to 126‐fold [50]. Despite the absence of glutamine–Q‐pocket interaction in the human p12 PIP motif, the measured binding affinities of *Ct* PolD4 and human p12 PIP peptides for their cognate PCNAs are broadly similar (*K*_D_ values of 22 and 38 μm, respectively), suggesting that interactions involving other amino acids in the *Ct* PolD4 PIP motif are weaker than those of human p12.

In almost all PIP motifs that have been characterised previously, including the archetypal p21^Cip1^ and human p12 PIPs, the PIP peptide adopts a 3_10_ helical conformation (Fig. 6B) between the conserved hydrophobic and aromatic residues, but the shortness of the distance between these residues in *Ct* PolD4 precludes this, giving rise to a compact 2‐fork plug binding motif spanning only six amino acids (^23^QSTLNF^28^) (Figs 5 and 6). This compact binding mode is not seen with any of the PIP motif–PCNA interactions that have been previously characterised at the structural level, or can it be confidently predicted for any of the known PIP motif–PCNA interactions that await structural characterisation [21]. The PolD4 PIP motif is one of at least three PIP or PIP‐like motifs found in the *C. thermophilum* Pol δ complex: in addition to the PolD4 PIP motif described here, the C‐terminal PIP motifs found in the human p125 and p66 proteins are well conserved in the fungal enzyme (D. Yang and S. MacNeill, unpublished data, summarised in Fig. 1). As noted above, the PolD1 PIP–PCNA interaction is likely to be a key feature of the processive Pol δ–PCNA complex while the roles of the PolD3–PCNA and PolD4–PCNA interactions remain unclear.

An important question to be addressed is whether the *C. thermophilum* PolD4 PIP motif functions as a PIP degron, as is the case for the human p12 PIP [32, 33]. Initial studies identified degron‐specific threonine and aspartate resides (termed the TD motif) between the conserved hydrophobic and aromatic residues of multiple PIP degrons, including human p21^Cip1^, Cdt1, Set8 and TDG, as well as a characteristic basic residue four amino acids downstream of the PIP motif (e.g. in the sequence ^144^QTSMTDFYHSKR ^155^ from human p21^Cip1^, basic residue Arg155 underlined) [35, 36]. Neither of these features is seen with PIP motifs that do not act as degrons. The *Ct* PolD4 PIP (^23^QSTLNF^28^) lacks the TD motif and a basic residue of four amino acids after Phe28, perhaps suggesting that the protein is not a target for CRL4^Cdt2^ in *C. thermophilum*. Inspection of the multiple‐sequence alignment of fungal PolD4 proteins in Fig. 3 reveals conserved basic amino acids three and six amino acids after Phe28, however. Unfortunately, although the PIP peptide (^21^KHQSTLNFKHRVTKP^35^) used in this study was 15 amino acids long and included both Arg31 and Lys34, no density was seen beyond Phe28, suggesting that residues 29–36 are not tightly bound to PCNA. Ultimately, further work *in vivo* will be required to determine whether the *Ct* PolD4 acts as a degron or not. The recent development of enhanced genetic tools [40] offers the possibility of manipulating the *C. thermophilum* genome to test the effects of mutating the PolD4 PIP motif and surrounding residues on PolD4 protein stability.

## Materials and methods

### Identification of *C. thermophilum* PCNA and PolD4 orthologues

Genes encoding *C. thermophilum* PCNA and PolD4 homologues were identified by BLAST searching using *S. pombe* Pcn1 (PCNA) and Cdm1 sequences as queries. To confirm splicing patterns, cDNAs for each gene were amplified from *C. thermophilum* cDNA (a generous gift of E. Hurt, University of Heidelberg) and sequenced. The sequences of both cDNAs (see Tables S1 and S2) have been submitted to the GenBank database (accession numbers: PCNA, GenBank: MW699350; PolD4, GenBank: MW699351).

### Plasmid construction

For recombinant expression in *E. coli* with a N‐terminal TEV cleavable His6 tag, the *C. thermophilum* PCNA ORF was synthesised in codon‐optimised form with flanking NcoI and NotI sites (see Table S3 for sequence) and cloned into plasmid pEHISTEV [51] to generate pEHISTEV‐Ct PCNA (Table S4).

For expression of GST–Ct PolD4‐PIP fusion proteins, a codon‐optimised synthetic DNA encoding four tandem repeats of the PolD4 amino acids 19–38 followed by a StrepII affinity tag (Table S5) was cloned as a BamHI‐NotI fragment into plasmid pGEX6P‐1 to generate plasmid pGEX6P1‐Ct PolD4‐4PIP‐Strep‖ (Table S4). Modified plasmids expressing GST–Ct PolD4‐PIP fusion proteins with one, two or three tandem PIP repeats (Table S6) were generated by deleting sequences from pGEX6P1‐Ct PolD4‐4PIP‐Strep‖ by *in vitro* mutagenesis (Q5 Site‐Directed Mutagenesis kit, New England Biolabs, Ipswich, MA, USA). Further mutagenesis of plasmid pGEX6P1‐Ct PolD4‐1PIP‐Strep‖ produced plasmids expressing GST–Ct PolD4‐PIP fusion proteins with a single copy of the PIP sequence but with extended N‐ or C‐terminal ends (or both): pGEX6P1‐Ct PolD4‐1PIP‐14‐38‐Strep‖, pGEX6P1‐Ct PolD4‐1PIP‐19‐43‐Strep‖ and pGEX6P1‐Ct PolD4‐1PIP‐14‐43‐Strep‖ (Table S4). The sequences of the primers used for *in vitro* mutagenesis are given in the Table S7.

### PCNA expression and purification

*Ct* PCNA was expressed from plasmid pEHISTEV‐Ct PCNA in *E. coli* Rosetta 2 (DE3) pLysS cells (Merck, Darmstadt, Germany) grown in LB medium containing chloramphenicol (34 μg·mL^−1^) and kanamycin (25 μg·mL^−1^). Expression was induced by the addition of IPTG to a final concentration of 1 mm. Induced cultures were grown at 30 °C for 6 h, before the cells were harvested by centrifugation at 1500 *g* for 10 min at 4 °C. Cell pellets were washed once in ice‐cold PBS before being resuspended in buffer A (50 mm Tris–HCl, pH 8.0, 150 mm NaCl and 20 mm imidazole) containing protease inhibitors (cOmplete™, Roche, Basel, Switzerland). The cells were lysed by sonication and the lysate was cleared by centrifugation at 118 000 *g* for 30 min at 4 °C. The soluble extract was then applied to a Ni‐NTA agarose (Qiagen, Hilden, Germany) purification column that had been pre‐equilibrated with buffer A. The column was washed with 25 column volumes (CV) buffer B (50 mm Tris–HCl, pH 8.0, 1 m NaCl and 20 mm imidazole) and bound protein eluted with 2.5 CV of Buffer C (50 mm Tris–HCl, pH 8.0, 250 mm NaCl and 250 mm imidazole). The elution fractions were concentrated and then subjected to cleavage by His6‐tagged TEV protease (1 : 10 protease:protein mixture) by overnight dialysis against 1 L of buffer A at 4 °C. The protein solution was then subjected to a second Ni‐NTA agarose column for removal of uncleaved His6‐PCNA protein along with the cleaved His6 tag and His6‐TEV protease. The flow through from the column was collected and subjected to size‐exclusion chromatography (SEC) using a HiLoad™ 26/600 Superdex™ 200 pg column in a buffer containing 50 mm Tris–HCl, pH 8.0 and 150 mm NaCl. The elution fractions from SEC were collected and analysed by SDS/PAGE. Fractions containing *Ct* PCNA were concentrated using a Amicon® Ultra‐15 Centrifugal Filter (30 kDa MWCO, Merck). The final concentration of *Ct* PCNA was measured by absorbance at 280 nm to be 14.4 mg·mL^−1^. A yield of 21.6 mg·L^−1^ *Ct* PCNA protein was obtained following the purification procedure.

### GST‐*Ct* PolD4‐PIP‐StrepII expression, purification and PCNA pull downs

GST‐*Ct* PolD4‐PIP‐StrepII proteins were expressed from pGEX6P‐Ct PolD4‐PIP plasmids (Table S4) in *E. coli* Rosetta 2 (DE3) pLysS cells (Merck) grown in 50 mL of LB medium containing chloramphenicol (34 μg·mL^−1^) and ampicillin (100 μg·mL^−1^). Expression was induced by the addition of IPTG to a final concentration of 0.1 mm. Induced cultures were grown at 37 °C for 4 h, before the cells were harvested by centrifugation at 3000 rpm for 10 min at 4 °C. Cell pellets were resuspended in 1.8 mL of buffer W (100 mm Tris–HCl pH 8.0, 150 mm NaCl and 1 mm EDTA) containing protease inhibitors (cOmplete™, Roche). The cells were lysed by sonication and the lysate was cleared by centrifugation at 16 060 *g* for 15 min at 4 °C. Soluble protein extract was then mixed with 100 μL of Strep‐Tactin® Sepharose® beads for 1 h at 4 °C. The beads were washed five times with 500 μL of buffer W before bound proteins were eluted with 1 mL of buffer BXT (100 mm Tris–HCl, pH 8.0, 150 mm NaCl, 1 mm EDTA and 50 mm biotin). Five hundred microlitre of the eluted material was incubated with 50 μL of Glutathione–Sepharose beads for 1 h at 4 °C. The beads were then washed five times with 500 μL buffer W containing 0.1% Triton X100, and 20 μL of protein‐bound beads were mixed with 200 μg of purified *Ct* PCNA for 30 min at 4 °C in a total volume of 500 μL of the same buffer. The beads were washed five times with 500 μL buffer W containing 0.1% Triton X100 at room temperature with each wash comprising 10 s of mixing with the buffer followed by centrifugation at 3000 rpm for 2 min. After the fifth wash, the beads were resuspended in 2× SDS/PAGE sample buffer, heated to 95 °C for 5 min and spun briefly before being subjected to SDS/PAGE.

### PolD4 PIP peptide

A *Ct* PolD4 PIP peptide spanning residues 21–35 (sequence: ^21^KHQSTLNFKHRVTKP^35^) was commercially synthesised (GenScript, Piscataway, New Jersey, USA) and obtained in lyophilised form at a final purity of 99.5%. The peptide was resuspended in 50 mm Tris–HCl, 50 mm NaCl, pH 8.0, at a concentration of 8 mg·mL^−1^ (4.4 mm).

### Isothermal calorimetry

ITC measurements were done using a MicroCal PEAQ‐ITC calorimeter (Malvern Panalytical, Malvern, Worcestershire, UK) with *Ct* PCNA protein and *Ct* PolD4 PIP peptide in 50 mm Tris–HCl, 50 mm NaCl, pH 8.0. Both the protein and peptide were subjected to filtration through a 0.22 μm syringe filter (Merck) before ITC measurements. For control titrations, buffer (50 mm Tris–HCl, 50 mm NaCl, pH 8.0) without protein was titrated with the *Ct* PolD4 PIP peptide (400 μm) at 25 °C. For the *Ct* PolD4 PIP peptide‐*Ct* PCNA titrations, approximately 300 μL of *Ct* PCNA protein solution (30 μm) was injected into the cell and then titrated with *Ct* PolD4 PIP peptide (400 μm) at 25 °C. A total of 19 injections were done for each titration assay (with a first injection of 0.2 μL, followed by 18 injections of 2 μL). Values from the control titrations were subtracted from the experimental titrations and the data fitted to a single‐site binding model (i.e. 1 peptide : 1 PCNA protomer) by MicroCal ITC analysis software (version 1.21). All measurements were performed in duplicate.

### Crystallisation, data collection and structure solution

Crystal screening of the apo‐protein used the PACT Premier™ screen (Molecular Dimensions, Holland, OH, USA) using 300 nL drops set up on an Gryphon crystallisation robot (ARI – Art Robbins Instruments, Sunnydale, CA, USA) resulted in crystal growth in 0.1 m PTCP buffer pH 5.0 and 25% PEG 1500. The crystals were of a size and quality that data could be collected directly from the screen and no additional cryoprotectant was required for data collection. The apo‐protein crystals diffracted to 2.34 Å. The crystals were found to be space group H3 with a single chain in the asymmetric unit, and the trimeric assembly generated by the crystallographic symmetry. Data collection statistics are shown in Table 1.

Soaking of the apo‐crystals with peptide (^21^KHQSTLNFKHRVTKP^35^) did not produce a complex of the two components and so co‐crystallisation screening was performed. Crystals grew in the JCSG+ screen (Molecular Dimensions), the precipitant containing 0.2 m calcium acetate, 0.1 m sodium cacodylate pH 6.5 and 40% PEG 300. Subsequent precipitant optimization to 0.2 m calcium acetate, 0.1 m sodium cacodylate pH 6.5 and 36% PEG 300 gave diffraction‐quality crystals. The precipitant was substituted with 20% glycerol for cryoprotection. The crystals diffracted to 2.45 Å and were found to be space group P1 with two trimers in the asymmetric unit. Data collection statistics are shown in Table 1.

All data were collected at 100 K in‐house on a Rigaku MM007HF Cu anode X‐ray generator (Rigaku, Tokyo, Japan) with a wavelength of 1.54178 Å and reflections were recorded on a Rigaku Saturn 944+ CCD detector. Data were processed using iMOSFLM [52] and scaled using AIMLESS [53].

The apo‐protein structure was solved by molecular replacement using MOLREP (CCP4) and using chain A of the homologous PDB entry PDB: 5TUP [44] as a starting model. Rounds of model building and refinement using COOT [54] and REFMAC5 [55] resulted in a model with an R and R_free_ of 21.3% and 28.0% respectively (see Table 1). The model was deposited in the Protein Data Bank under accession number PDB: 7O1E.

The structure of the protein–peptide complex was solved by molecular replacement using MOLREP [56] and with the apo‐protein structure 7O1E as the search model. Rounds of model building and refinement using COOT and REFMAC5 resulted in a model with an *R* and *R*_free_ of 22.0% and 26.2% respectively (see Table 1). The model was deposited in the Protein Data Bank under accession number PDB: 7O1F.

## Conflict of interest

The authors declare no conflict of interest.

## Author contributions

SM conceived the study. DY, MA and SM performed the experimental work. SM drafted the manuscript with the assistance of DY and MA. All authors read, revised and approved the final manuscript.

## Supporting information


**Table S1.** *Chaetomium thermophilum* PCNA gene structure.
**Table S2.** *Chaetomium thermophilum* POLD4 gene structure.
**Table S3.** Codon‐optimised synthetic DNA‐encoding PCNA.
**Table S4.** Plasmids used in this work.
**Table S5.** Codon‐optimised synthetic DNA‐encoding four PolD4 PIP motifs (amino acids 19–38) with C‐terminal StrepII tag for GST fusion.
**Table S6.** Sequences of single and multiple *Ct* PolD4‐PIP constructs.
**Table S7.** Oligonucleotide primers used in this work.

## Data Availability

*3D macromolecular structures*: The structural data that support these findings are openly available in the RSCB PDB at https://www.rcsb.org/structure/7O1E (PCNA) and https://www.rcsb.org/structure/7O1F (PCNA‐PIP motif peptide). *Nucleotide sequence data*: The nucleotide sequence data that support the findings in this study are openly available in GenBank at https://www.ncbi.nlm.nih.gov/nuccore/MW699350 (PCNA) and https://www.ncbi.nlm.nih.gov/nuccore/MW699351 (PolD4). *Data available within the article and/or the supplementary material*: The data that support the findings of this study are available in Figs 1, 2, 3, 4, 5, 6 and Table 1 and/or the Supplementary Material of this article.
