# Supplementary material for: Non‐canonical binding of the Chaetomium thermophilum PolD4 N‐terminal PIP motif to PCNA involves Q‐pocket and compact 2‐fork plug interactions but no 310 helix
Source: FEBS J. 2022 Aug 22;290(1):162–75. doi: 10.1111/febs.16590 (PMC10087552; doi:10.1111/febs.16590)
Supplement: Supplementary file 1 — Table S1. Chaetomium thermophilum PCNA gene structure. Table S2. Chaetomium thermophilum POLD4 gene structure. Table S3. Codon‐optimised synthetic DNA‐encoding PCNA. Table S4. Plasmids used in this work. Table S5. Codon‐optimised synthetic DNA‐encoding four PolD4 PIP motifs (amino acids 19–38) with C‐terminal StrepII tag for GST fusion. Table S6. Sequences of single and multiple Ct PolD4‐PIP constructs. Table S7. Oligonucleotide primers used in this work. [file FEBS-290-162-s001.pdf]

Yang, Alphey and MacNeill (2022)

Supplementary information

Table S1: *Chaetomium thermophilum* POLD4 gene structure
ATGCCTCCCACCCGCCGCTCAACCAGATCCTCCTCCACTGCCACCGGCAGCGGCAGTACAAAGCACCAATCAACCCTCAAC<br>TTTAAGCACCGCGTCACCAAGCCTGTCTCCTCCACAGCCAAAGACACCAAAGAAAAAGAAACCAAATCCCCCGCCCGGGCG<br>AAGAAGGAAATCATTGTCCCTGCTCCCGAACCTGCCCCCAAGGAGGAGCAGGATGAAGTAAAGGAGGAGGAAACTAATGAG<br>GAAGTTAAGGAGGTGGAGGAGAAGGAGGTCAAAAATGCGAGGAGTGATCCTGAAAGCCGCGCCGCCAAAGTGACGAACGCC<br>CAGATTGAGAAGTACTGGAAGGGGATTGAGGCGTCGCGCATGGCGAAGGAGTTGCATCGGAAGCATGGGCAGGGACTAAGT<br>ACGGAGGAGAAGGTGCTGAGGTATTTTGATGTTAGTTCGCAGTATGGG**GTACGTCCTTTTACCCCCCCTCTCTCTCTTTT**<br>**TCTTTTTTTTTTTTTTTTGTTTTCTTTACGGTGGAGGGATTTTTTTTTAAGAACTAACGATATGACAG**CCCTGTGTTGGC<br>ATTACTCGGCTTAGACGCTGGCAGCGTGCTGAGAGATTGGGCCTCAACCCGCCAATTGAGGTGTTGGCGGTGTTGATGAAG<br>GAGAAGGCCAAAGGCAATGAGGGGATTCAGAGAGCTCATATGGATGAATTGTTGAGCTCGACTGCTGTGGGGTCCGTGTGA
Notes: Start and stop codons underlined. Intron sequence shown in red. GenBank accession number for PolD4 cDNA sequence: MW699351.

Table S2: *Chaetomium thermophilum* PCNA gene structure
ATGTTAGAAGCACGGTTGGAGCAGGCCAGCATCCTGAAGAAAGTGGTTGACGCCATCAAGGATCTGGTCCAGGACTGCAAC<br>TTCGACTGCAATGATAGCGGTATCGCCCTTCAAGCTATGGACAACAGCCATGTCGCTCTCGTGTCCATGATGCTTAAGGCC<br>GAAGGCTTTTCCCCCTACCGCTGCGATCGTAACATCGCGCTCGGCGTAAATCTCACTTCTCTCACCAAAGTGCTGCGTGCC<br>GCCCAAAACGAAGATATTCTGACTCTCAAGGCCGAGGACGCCCCTGATGTCCTGAATCTGGTCTTTGAGAGTTCTGAGACT<br>GATCGCATCTCCGAGTACGACCTTAAGCTCATGGACATTGACCAAGAGCACCTGGGTATTCCGGAGACCGAATATGCTGCC<br>ACTATTACCATGCCATCCAATGAGTTCAAGCGTATCACGACCGACTTGATGGCCATGTCGGAATCTG**GTACGATTCTCCTT**<br>**GAGCACACCATTTAAAAATTCTTAAAAGGCTTTCCGCTAACACGCCAATTTCAG**TAACCATCGAGGCTAACAAAGACGGCG<br>TCAAGTTCTCTTGCCAGGGTGACATTGGCAATGGGTCTGTGACGCTTCGTCAGCATACCAACGTCGAGAAGCCTAACGAGT<br>CGATTGAAATCGAGCTCAGCGAGCCCGTCTCCCTGACTTTCTCTCTCAAGTACCTTGTCAACTTCTGCAAGGCCTCGGCGC<br>TCTCGAACACTGTGAAGATCTGCCTTTCCAACGAGGTACCCCTGCTGGTTGAATACAGCTTGGGTGGCAGCAGCTACCTGC<br>GCTTCTACCTTGCTCCGAAG**GTATGCTGTTTGGTCTTTGTATGTTGTTTTGTTCGATGACCAGTTGGCTAACTGTGTATAG**<br>ATTGGTGATGATGAGTAA
Notes: Start and stop codons underlined. Intron sequences shown in red. GenBank accession number for PCNA cDNA sequence: MW699350.

Table S3: Codon-optimised synthetic DNA encoding PCNA
CCATGGCTCTGGAAGCACGTCTTGAGCAAGCTAGTATTTTGAAAAAGGTTGTGGATGCTATTAAAGATTTAGTACAGGACT<br>GTAATTTTGACTGTAATGACTCAGGGATTGCTTTACAAGCAATGGATAATTCGCACGTGGCATTGGTGAGTATGATGTTAA<br>AGGCTGAGGGCTTTTCGCCATATCGTTGTGATCGTAATATCGCCTTAGGTGTTAATTTGACCTCACTTACTAAAGTACTTC<br>GCGCTGCTCAAAACGAAGATATCTTGACCCTGAAGGCGGAAGATGCTCCTGACGTTCTGAACTTAGTTTTCGAATCTTCCG<br>AAACCGACCGCATTTCAGAATATGATCTGAAACTTATGGACATCGACCAGGAACACTTGGGTATCCCTGAAACAGAGTACG<br>CAGCTACTATTACGATGCCTTCGAATGAGTTCAAGCGCATCACAACCGACTTAATGGCTATGAGTGAAAGCGTTACGATTG<br>AGGCCAATAAAGATGGTGTCAAGTTTAGTTGTCAAGGAGATATTGGCAACGGTTCGGTCACATTACGTCAACATACCAATG<br>TTGAAAAACCAAATGAATCGATCGAGATTGAACTTAGTGAGCCTGTTAGTCTTACTTTTAGTCTGAAGTATCTTGTGAATT<br>TTTGCAAGGCGTCAGCGTTAAGCAATACAGTGAAAATCTGCTTATCAAATGAAGTCCCACTTTTAGTGGAATACAGTTTGG<br>GTGGCTCAAGCTACTTACGCTTCTATTTAGCGCCCAAGATTGGAGACGATGAATAATGAGCGGCCGC
Notes: NcoI and NotI sites used for cloning underlined.

Table S4: Plasmids used in this work		
**Plasmid name**	**Expressed protein**	**Source/reference**
pEHISTEV	N/A	{Liu, 2009 #4414}
pEHISTEV-CtPCNA	His6-TEV-CtPCNA	This study
pGEX6P-1	GST	Cytiva
pGEX6P1-CtPolD4-1PIP-StrepII	GST-CtPolD4-1PIP-StrepII	This study
pGEX6P1-CtPolD4-2PIP-StrepII	GST-CtPolD4-2PIP-StrepII	This study
pGEX6P1-CtPolD4-3PIP-StrepII	GST-CtPolD4-3PIP-StrepII	This study
pGEX6P1-CtPolD4-4PIP-StrepII	GST-CtPolD4-4PIP-StrepII	This study
pGEX6P1-CtPolD4-1PIP-14-38-StrepII	GST-CtPolD4-1PIP-14-38-StrepII	This study
pGEX6P1-CtPolD4-1PIP-19-43-StrepII	GST-CtPolD4-1PIP-19-43-StrepII	This study
pGEX6P1-CtPolD4-1PIP-14-43-StrepII	GST-CtPolD4-1PIP-14-43-StrepII	This study

Table S5: Codon-optimised synthetic DNA encoding four PolD4 PIP boxes (amino acids 19-38) with C-terminal StrepII tag for GST fusion
**GGATCC**AGTACCAAGCACCAGTCCACGTTGAATTTTAAGCACCGCGTAACAAAACCTGTATCAAGTTCTACGAAGCATCAG TCAACTTTAAATTTCAAACATCGCGTGACGAAGCCAGTGTCCTCATCCACAAAACACCAAAGTACGCTTAATTTTAAGCAC CGTGTTACCAAACCGGTCTCATCTTCGACCAAACATCAGAGTACCTTAAACTTTAAGCATCGTGTGACTAAGCCGGTGAGT AGTTGGTCCCACCCGCAATTTGAAAAATGA**GCGGCCGC**
Notes: BamHI and NotI sites shown in bold. Stop codon underlined.

Table S6: Sequences of single and multiple Ct PolD4-PIP constructs	
CtPolD4-1PIP	GSSTKHQSTLNFKHRVTKPVSS**WSHPQFEK**
CtPolD4-2PIP	GSSTKHQSTLNFKHRVTKPVSSSTKHQSTLNFKHRVTKPVSS **WSHPQFEK**
CtPolD4-3PIP	GSSTKHQSTLNFKHRVTKPVSSSTKHQSTLNFKHRVTKPVSS STKHQSTLNFKHRVTKPVSS**WSHPQFEK**
CtPolD4-4PIP	GSSTKHQSTLNFKHRVTKPVSSSTKHQSTLNFKHRVTKPVSS STKHQSTLNFKHRVTKPVSSSTKHQSTLNFKHRVTKPVSS**WSHPQFEK**
CtPolD4-1PIP-14-38	GSATGSGSTKHQSTLNFKHRVTKPVSS**WSHPQFEK**
CtPolD4-1PIP-19-43	GSSTKHQSTLNFKHRVTKPVSSTAKDT**WSHPQFEK**
CtPolD4-1PIP-14-43	GSATGSGSTKHQSTLNFKHRVTKPVSSTAKDT**WSHPQFEK**
Notes: The initial two amino acids (GS) are encoded by the BamHI site used for cloning. The PIP sequence (underlined) corresponds to amino acids 19-38 of CtPolD4. The final three sequences extend the PIP by five amino acids at the N-terminus (residues 14-38), C-terminus (19-43) or both (14-43).The C-terminal StrepII tag is shown in bold.	

Table S7: Oligonucleotide primers used in this work	
Primers for mutagenesis of pGEX6P1-CtPolD4-4PIP-StrepII	
CtPolD4-IVM-3PIP-FOR	5′-TCTACGAAGCATCAGTCAACTTTAAATTTCAAACATCG-3′
CtPolD4-IVM-2PIP-FOR	5′-TCGACCAAACATCAGAGTACCTTAAACTTTAAGC-3′
CtPolD4-IVM-1PIP-FOR	5′-TCCACAAAACACCAAAGTACGCTTAATTTTAAGC-3′
CtPolD4-IVM-REV	5′-GGATCCCAGGGGCCCCTG-3′
Primers for mutagenesis of pGEX6P1-CtPolD4-PIP-StrepII	
CtPolD4-14-18-FOR	5′-CAGCGGCTCGACCAAACATCAGAGTACCTTAAACTTTAAGC-3′
CtPolD4-14-18-REV	5′-CCGGTGGCGGATCCCAGGGGCCCCTG-3′
CtPolD4-39-43-FOR	5′-AGACACCTGGTCCCACCCGCAATTT-3′
CtPolD4-39-43-REV	5′-TTGGCTGTACTACTCACCGGCTTAGTC-3′
